# Plasma microRNA miR-26b as a potential diagnostic biomarker of degenerative myelopathy in Pembroke welsh corgis

**DOI:** 10.1186/s12917-019-1944-3

**Published:** 2019-06-10

**Authors:** Kohei Nakata, Kazuki Heishima, Hiroki Sakai, Osamu Yamato, Yu Furusawa, Hidetaka Nishida, Sadatoshi Maeda, Hiroaki Kamishina

**Affiliations:** 1 The United Graduate School of Veterinary Sciences, Gifu University, 1-1 Yanagido, Gifu, 501-1193 Japan; 2 The United Graduate School of Veterinary Sciences and Center for Highly Advanced Integration of Nano and Life Sciences, Gifu University, 1-1 Yanagido, Gifu, 501-1193 Japan; 3 Joint Faculty of Veterinary Medicine, Kagoshima University, 1-21-24 Kohrimoto, Kagoshima, 890-0065 Japan

**Keywords:** Canine, Degenerative myelopathy, microRNA, Diagnostic biomarker, Neurology

## Abstract

**Background:**

Degenerative myelopathy (DM) is a progressive neurodegenerative disease frequently found in Pembroke Welsh Corgis (PWCs). Most DM-affected PWCs are homozygous for the mutant superoxide dismutase 1 (*SOD1*) allele; however, the genetic examination for the SOD1 mutation does not exclusively detect symptomatic dogs. In order to identify novel biomarkers, the plasma microRNA (miRNA) profiles of PWCs with DM were investigated.

**Results:**

Quantification of the plasma levels of 277 miRNAs by an RT-qPCR array identified 11 up-regulated miRNAs and 7 down-regulated miRNAs in DM-affected PWCs from those in wild-type SOD1 PWCs. A pathway analysis identified 3 miRNAs: miR-26b, miR-181a, and miR-196a, which potentially regulate several genes associated with SOD1. In order to validate the diagnostic accuracy of the candidate miRNAs in the aged PWC population, candidate miRNAs in plasma were measured by RT-qPCR and a receiver operating characteristic (ROC) curve analysis was performed. miR-26b had the largest area under the ROC curve for distinguishing DM PWCs from healthy PWCs (sensitivity, 66.7%; specificity, 87.0%). The plasma level of miR-26b was significantly higher in the DM group than in the healthy control group. A positive correlation was observed between increases in the plasma level of miR-26b and disease progression.

**Conclusions:**

These results suggest that plasma miR-26b is a potential novel diagnostic biomarker of DM.

**Electronic supplementary material:**

The online version of this article (10.1186/s12917-019-1944-3) contains supplementary material, which is available to authorized users.

## Background

Canine degenerative myelopathy (DM) is an adult-onset progressive neurodegenerative spinal cord disorder that occurs in multiple dog breeds including Boxers, German Shepherds, and Pembroke Welsh Corgis (PWCs) [1, 2]. The clinical signs of DM are characterized by general proprioceptive ataxia and upper motor neuron spastic paresis of the pelvic limbs, which gradually progress to flaccid tetraplegia and brain stem signs. If dogs are not euthanized during the early stages and the disease is allowed to progress, affected dogs exhibit signs of respiratory muscle paralysis and ultimately die from respiratory failure approximately 3 years after disease onset [2, 3]. Previous studies identified two types of single nucleotide mutations (c.118G > A, c.52A > T) in DM-affected dogs that lead to alterations in a single amino acid of SOD1 (E40K and T18S, respectively) [4, 5]. Dogs with DM consistently display the aggregation of the mutant SOD1 protein in their spinal neurons [4, 6]. Although the pathomechanisms of DM have not yet been fully elucidated, it is hypothesized that neural degeneration in the DM spinal cord is caused by a gain of toxic function of mutant SOD1 because mutant SOD1 protein has been reported to retain full enzymatic activity [7, 8]. Based on similar clinical signs, neuropathological findings, and the involvement of the SOD1 mutation, DM is regarded as a naturally-occurring model of amyotrophic lateral sclerosis (ALS) [2, 5, 9].

A definitive diagnosis of DM can only be reached by a post-mortem histopathological examination of the spinal cord [2]. A pre-mortem diagnosis is currently based on three steps: pattern recognition of the progression of clinical signs, genetic testing for the SOD1 mutation, and eliminating other diseases affecting the spinal cord [2, 10]. Although quantification of CSF and serum pNF-H concentration has been reported as a diagnostic tool for DM [11], it is difficult to differentiate DM from other central and peripheral axonopathies based solely on the measurement of this particular biomarker. Although homozygosity for the E40K SOD1 mutation has been identified as a major risk factor for DM, many dogs homozygous for the mutation do not develop clinical signs [12, 13]. In addition, the clinical signs of DM may mimic other progressive spinal cord diseases, some of which may also co-exist with DM, thereby confounding a clinical diagnosis. The development of diagnostic biomarkers of DM is important in order to more accurately differentiate DM from other neurological diseases with a similar clinical presentation.

MicroRNAs (miRNAs) are small (18–25 nucleotides) non-coding RNAs that play important regulatory roles by targeting messenger RNAs (mRNAs) for cleavage or translational repression [14]. Due to their regulatory functions in different cellular processes including cell growth, differentiation, cell proliferation, and apoptosis, miRNAs act as key regulators of various biological functions in the nervous system, such as synaptic plasticity, neuronal differentiation, and neuroinflammation [15]. Since the expression of some miRNAs is specific to tissues or biological stages, changes in these miRNA concentrations in the central nervous system lead to the etiology and progression of neurodegenerative disorders [16, 17]. Some miRNAs are found in biological fluids such as blood, urine, and cerebrospinal fluid and are encapsulated in microvesicles as a relatively stable form [18]. Therefore, miRNAs have potential as novel diagnostic biomarkers and predictors of the prognosis and therapeutic effects of some neurodegenerative diseases, such as ALS [19–21].

The aim of the present study was to identify potential candidates as diagnostic biomarkers of DM. We initially compared the plasma miRNA profile of dogs with DM with that of wild-type controls using a quantitative reverse transcription polymerase chain reaction (RT-qPCR) miRNA array to search for candidate diagnostic biomarkers of DM. We then evaluated the diagnostic accuracy of the selected miRNAs by RT-qPCR in an aged PWC population.

## Methods

### Clinical samples

In order to compare the plasma miRNA profile of dogs with DM with that of healthy controls, we included four DM-affected PWCs with homozygous *SOD1* mutation (*c.118G > A*) and four wild-type PWCs. The diagnostic accuracy of the selected miRNAs was evaluated in 18 DM-affected PWCs and 46 healthy control PWCs. PWCs were diagnosed with DM at the Animal Medical Center of Gifu University according to the following criteria: clinical signs consistent with DM (adult onset, slow progression, and non-painful paraparesis progressing to tetraplegia) [2, 10], unremarkable findings on spinal imaging with magnetic resonance imaging (MRI) (0.4-Tesla APERTO Eterna, Hitachi), cerebrospinal fluid (CSF) analyses, and genetic testing proving homozygosity for the *SOD1 c.118G > A* missense mutation (A/A) [12]. The disease stage was classified into four clinical stages as previously described [2, 10]. Briefly, the clinical characteristics of DM stages were as follows: Stage 1, general proprioceptive ataxia and upper motor neuron paraparesis; Stage 2, non-ambulatory paraparesis to paraplegia; Stage 3, lower motor neuron paraplegia to thoracic limb weakness; and Stage 4, lower motor neuron tetraplegia and brain stem signs. We obtained control blood samples from private veterinary clinics; these dogs were healthy PWCs that had visited hospitals for healthy checkup or vaccination and had no specific disease, were age-matched with DM-affected PWCs, and had various *SOD1* genotypes: A/A homozygotes, A/G heterozygotes, and G/G homozygotes. In addition, five PWCs with intervertebral disc herniation (IVDH) were included as disease controls. These PWCs presented with the acute onset of paraparesis or neck pain and spinal cord compression by herniated intervertebral discs confirmed by MRI or computed tomography (Asteion Super 4; Toshiba, Tochigi, Japan) with myelography.

### Sample preparation, miRNA extraction, and reverse transcription

Blood samples were collected in disodium ethylenediaminetetraacetate tubes and centrifuged at 1500×*g* at 4 °C for 20 min, after which plasma was separated and frozen at − 80 °C. Total plasma RNA was extracted from 200 μL of plasma using an miRNeasy Serum/Plasma Kit (QIAGEN). In the RT-qPCR array, 6.3 × 10^8^ copies of synthetic *Caenorhabditis elegans* mir-39 (miRNeasy Serum/Plasma Spike-In Control, QIAGEN) were added to each plasma sample in order to normalize and monitor extraction efficiency. Total RNA was reverse-transcribed to complementary DNA (cDNA) using a miScript II RT Kit (QIAGEN) and thermal cycler system (TaKaRa Thermal Cycler Dice, Takara). All protocols were performed in accordance with the manufacturers’ standard instructions.

### PCR array

Generated cDNA was preamplified using an miScript PreAMP PCR Kit (QIAGEN) and the thermal cycler system under the following thermal conditions: an initial activation step at 95 °C for 15 min, followed by 2 cycles of denaturation at 94 °C for 30 s, annealing at 55 °C for 60 s, extension at 70 °C for 30 s, and then 10 cycles of denaturation at 94 °C for 30 s and annealing/extension at 60 °C for 3 min. PCR arrays were applied to screen candidate miRNAs by using an miScript SYBR Green PCR Kit (QIAGEN) and miScript miRNA PCR Array Dog miRNome (QIAGEN), which carry 277 probes to detect canine miRNAs and cel-miR-39-3p probes as a spike-in control. Fluorescent PCR products were detected using a real-time PCR detection system (TaKaRa Thermal Cycler Dice Real Time System TP800, Takara) under the following conditions: an initial denaturation step at 95 °C for 15 min, followed by 40 cycles of denaturation at 95 °C for 15 s, annealing at 55 °C for 30 s, and extension at 70 °C for 30 s. After assessing threshold cycle (Ct) values, the relative expression levels of miRNA were calculated by the ∆∆Ct method using two controls, cel-miR-39-3p served as the spike-in control and miR-16 served as the normalization control [21, 22]. The criteria for up-regulated miRNAs were set as a fold change of more than 2.0 and down-regulated miRNAs were set as a fold change of less than 0.5 with a statistical cut-off of *P* < 0.05 using the Mann-Whitney *U*-test.

### Pathway analysis

The potential target genes of dysregulated miRNAs were identified using TargetScan database version 7.1 [23]. We collected the signaling pathways of *SOD1* from the WikiPathway database [24]. We integrated and illustrated the pathway from dysregulated miRNA to *SOD1* using a bioinformatics software platform (Cytoscape ver.3.4.0, National Resource for Network Biology). The miRNAs suggested to be associated with the pathogenesis of DM were selected as the candidate biomarkers.

### Validation of candidate miRNAs

After we obtained cDNA from plasma samples, we quantified the plasma levels of candidate miRNAs in duplicate using the miScript SYBR Green PCR Kit under the same conditions described above. The primers (miScript Primer Assays, QIAGEN) for target miRNAs were miR-26b (catalogue number MS00030205), miR-181a (MS00029715), and miR-196a (MS00029918). The primers to normalize control miRNAs were miR-16 (MS00037373) and cel-miR-39-3p (MS00019789). The 2^-∆Ct^ method was used to calculate the relative quantities of candidate miRNAs.

### Statistical analyses

Comparisons of two groups were performed by the Mann-Whitney *U*-test. Multiple group comparisons were assessed by the chi-squared test, Kruskal-Wallis test, and Steel-Dwass test. Spearman’s correlation coefficient was used for comparisons and estimations of correlations between miRNA expression levels and age or the disease stage. The receiver operating characteristic (ROC) curve analysis with the area under the curve (AUC) was performed in order to assess the diagnostic accuracy of candidate miRNA levels and the cut-off value to diagnose DM. Optimal cut-off values were selected for each candidate miRNA based on the highest Youden index. The sensitivity and specificity of each miRNA were calculated based on the optimal cut-off value. A *P* value of less than 0.05 was considered to be significant. All statistical calculations were performed with statistical software (JMP ver.13.2.0, SAS Institute Japan Ltd.).

## Results

### Sample characteristics

The characteristics of all samples are shown in Table 1. In miRNA microarray analyses, we included four DM-affected PWCs and four wild-type PWCs. In validation analyses, 18 DM-affected PWCs, 46 healthy control PWCs, and five IVDH PWCs as disease control were included. DM-affected PWCs and healthy control PWCs included PWCs whose plasma samples were used in microarray analysis but the miRNAs were re-extracted. At the time of sample collection, all DM-affected PWCs were diagnosed clinically. Three of four DM dogs in the miRNA microarray, except for one (DM 2), and seven of 18 DM dogs in the validation analysis were definitively diagnosed with DM by a histopathological examination during the study period. In the miRNA microarray, three dogs (DM 1–3) were classified as stage 4 and one (DM 4) as stage 3. In the validation analysis, we classified five dogs as stage 4, four dogs as stage 3, five dogs as stage 2, and four dogs as stage 1. There was no significant age bias among the three groups (*P* = 0.2523); however, the healthy control group had a significantly larger number of females than the DM and IVDH groups (*P* = 0.0007).

**Table 1:** Characteristics of clinical samples

		miRNA microarray		Validation analysis							
		DM ^a^	Wild-type	DM	HC ^b^				IVDH ^c^		
					Total	A/A	A/G	G/G	Total	A/A	A/G
Number	Total	4	4	18	46	21	21	4	5	4	1
	Stage 1	0	–	4	–	–	–	–	–	–	–
	Stage 2	0	–	5	–	–	–	–	–	–	–
	Stage 3	1	–	4	–	–	–	–	–	–	–
	Stage 4	3	–	5	–	–	–	–	–	–	–
Gender	Male	1	3	10	11	4	4	3	5	4	1
	Female	3	1	8	35	17	17	1	0	0	0
Age in months	Median	159	151	159	140	133	155	152	162	142	163
	Range	142–161	100–173	110–187	89–194	94–178	89–194	100–173	116–173	116–173	–

^a^Degenerative myelopathy

^b^Healthy control

^c^Intervertebral disc herniation

^d^Superoxide dismutase 1

### Comparison of plasma miRNA profiles between DM and wild-type PWCs

**Fig. 1:**
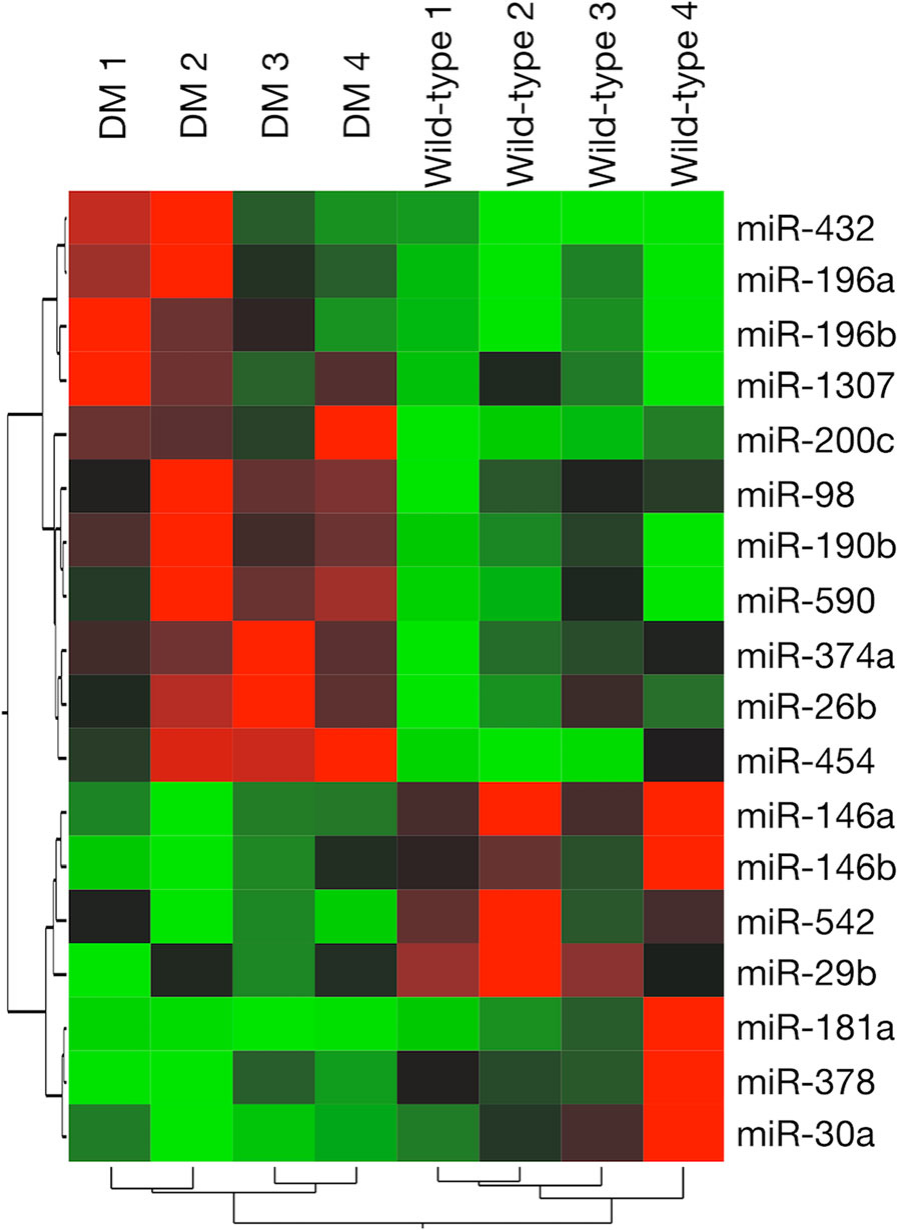
Heat map representation of 18 dysregulated plasma microRNAs (miRNAs) in degenerative myelopathy (DM) and normal Pembroke Welsh Corgis (PWCs). Up-regulated miRNAs are shown in red and down-regulated miRNAs are in green

In order to detect dysregulated plasma miRNAs in DM PWCs, we performed an RT-qPCR array on DM-affected and wild-type PWCs. We excluded 64 miRNAs that showed a non-specific reaction by detecting non-exponential curves and 11 miRNAs that were undetectable in all samples; therefore, we quantified 202 out of 277 canine miRNAs in plasma levels using the RT-qPCR array (Additional file 1: Table S1 and Additional file 2: Table S2). Among the remaining 201 miRNAs, excluding miR-16 as a normalization control, we detected 11 up-regulated miRNAs and 7 down-regulated miRNAs in DM-affected PWCs relative to those in wild-type PWCs (Fig. 1 and Table 2).

**Table 2:** Dysregulated microRNAs using a quantitative reverse transcription polymerase chain reaction array analysis in Pembroke Welsh Corgis with degenerative myelopathy

Differential expression type	miRNA	Fold Change	*P* value
Up-regulation	miR-26b	2.512	0.043
	miR-98	2.114	0.021
	miR-190b	5.136	0.021
	miR-196a	12.011	0.020
	miR-196b	12.937	0.042
	miR-200c	6.389	0.021
	miR-374a	2.237	0.021
	miR-432	23.988	0.018
	miR-454	6.025	0.043
	miR-590	4.713	0.043
	miR-1307	4.188	0.043
Down-regulation	miR-29b	0.416	0.021
	miR-30a	0.390	0.028
	miR-146a	0.378	0.019
	miR-146b	0.288	0.043
	miR-181a	0.143	0.021
	miR-378	0.160	0.021
	miR-542	0.221	0.043

### Pathway analysis

**Fig. 2:**
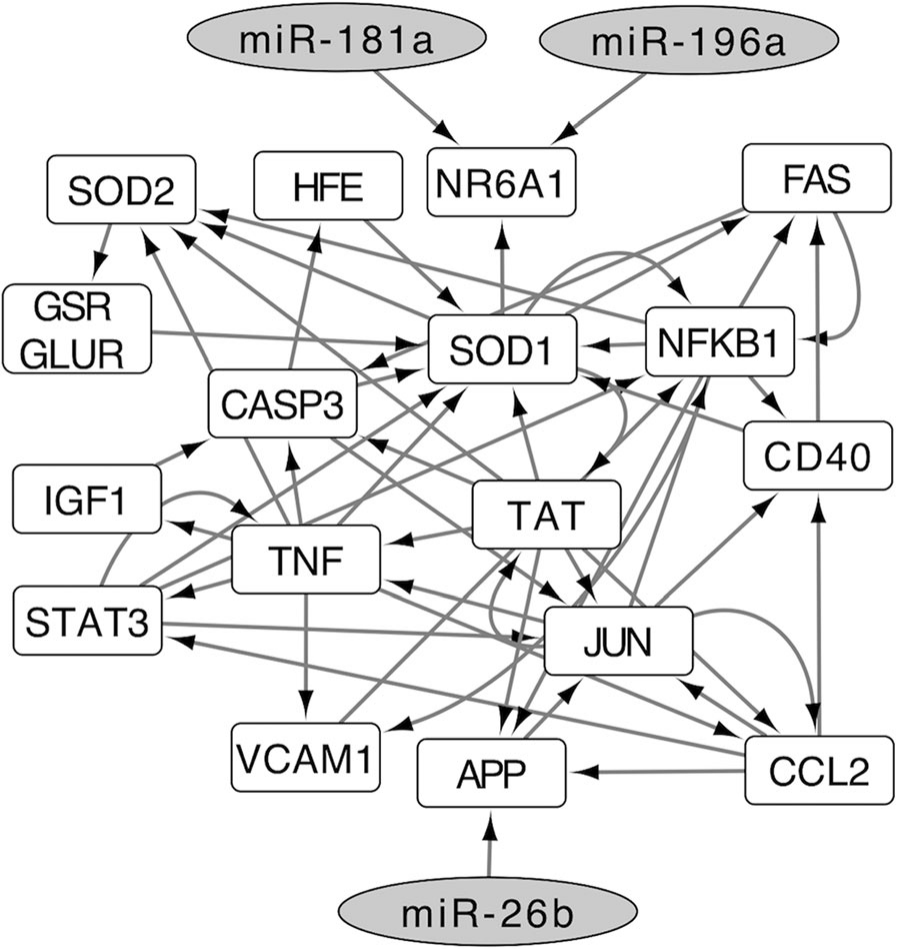
Interrelated networks of genes possibly associated with degenerative myelopathy (DM) and dysregulated microRNAs (miRNAs) in DM-affected Pembroke Welsh Corgis (PWCs)

In order to estimate the relationship between the miRNAs detected and the pathogenesis of DM, we performed a pathway analysis. We initially searched for putative mRNA targets of the 18 dysregulated miRNAs by using the TargetScan database with context++ scores < −0.40, which is based on compensatory binding in the 3′ of miRNAs (Additional file 3: Table S3). We subsequently searched for genes that were potentially associated with the pathogenesis of DM based on previous studies and detected four signaling pathways: the Oxidative Stress pathway, TNF-alpha NF-kB Signaling Pathway, Apoptosis Modulation by HSP70, and Amyotrophic lateral sclerosis SOD1 - CHAMP28 MODEL [25–28]. We then overlaid the above signaling pathways and dysregulated miRNA-mRNA interactions and constructed the miRNA pathway related to the pathogenesis of DM (Fig. 2). According to the constructed pathway analysis, we selected three miRNAs: miR-26b, miR-181a, and miR-196a, as candidate diagnostic biomarkers of DM.

### Validation analysis using RT-qPCR

**Fig. 3:**
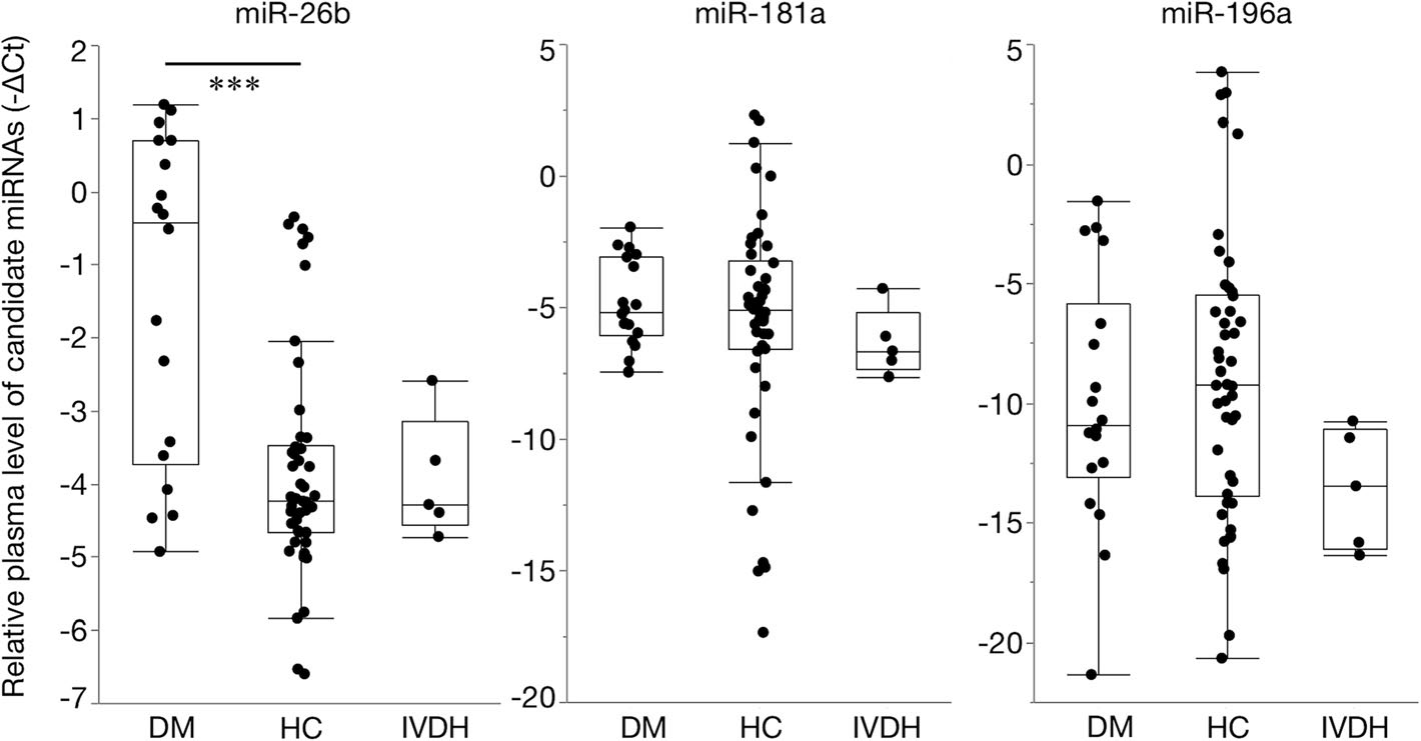
Boxplots of candidate plasma microRNAs (miRNAs) in degenerative myelopathy (DM)-affected Pembroke Welsh Corgis (PWCs), healthy control (HC) PWCs, and intervertebral disc herniation (IVDH)-affected PWCs (Steel-Dwass test; *** *P* < 0.001)

**Fig. 4:**
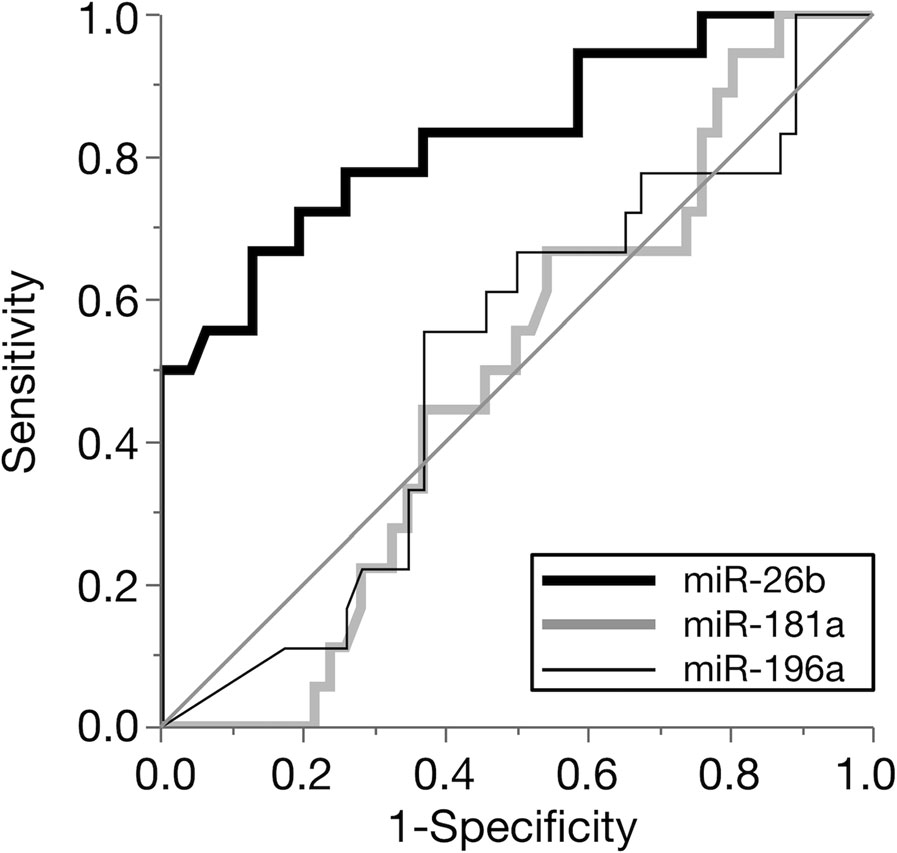
Receiver operating characteristic (ROC) curve of candidate plasma microRNAs (miRNAs) for the detection of degenerative myelopathy (DM)-affected Pembroke Welsh Corgis (PWCs) among 64 aged healthy PWCs

Based on the evaluation of the diagnostic accuracy of the candidate miRNAs (Additional file 4: Table S4), the relative plasma levels of miR-26b were higher in the DM group than in the healthy control and IVDH groups. A significant difference was detected between the DM and healthy control groups. In contrast, the relative plasma levels of miR-181a and miR-196a were not significantly different between any groups (Fig. 3). The plasma levels of each candidate miRNA did not correlate with age and there were no significant differences in gender. A ROC curve analysis revealed that miR-26b had the highest AUC value for discriminating DM-affected PWCs from healthy control PWCs (Fig. 4 and Table 3). Therefore, we selected miR-26b as a diagnostic biomarker of DM and set the cut-off value as 2^-∆Ct^ = 0.1996 based on the ROC curve analysis. At this cut-off value, the plasma level of miR-26b in the IVDH group did not exceed the threshold to diagnose DM.

**Table 3:** Receiver operating characteristic curve analysis

candidate miRNA	AUC ^a^	95% CI ^b^	Sensitivity	Specificity	Cut-off
miR-26b	0.8291	0.637–0.908	0.6667	0.8696	0.1996
miR-181a	0.4903	0.351–0.636	0.9444	0.1957	0.1610
miR-196a	0.5163	0.393–0.702	0.5556	0.6304	0.0006

^a^Area under the curve

^b^95% confidence interval

### Relationships between candidate miRNAs and the SOD1 allele or DM staging

**Fig. 5:**
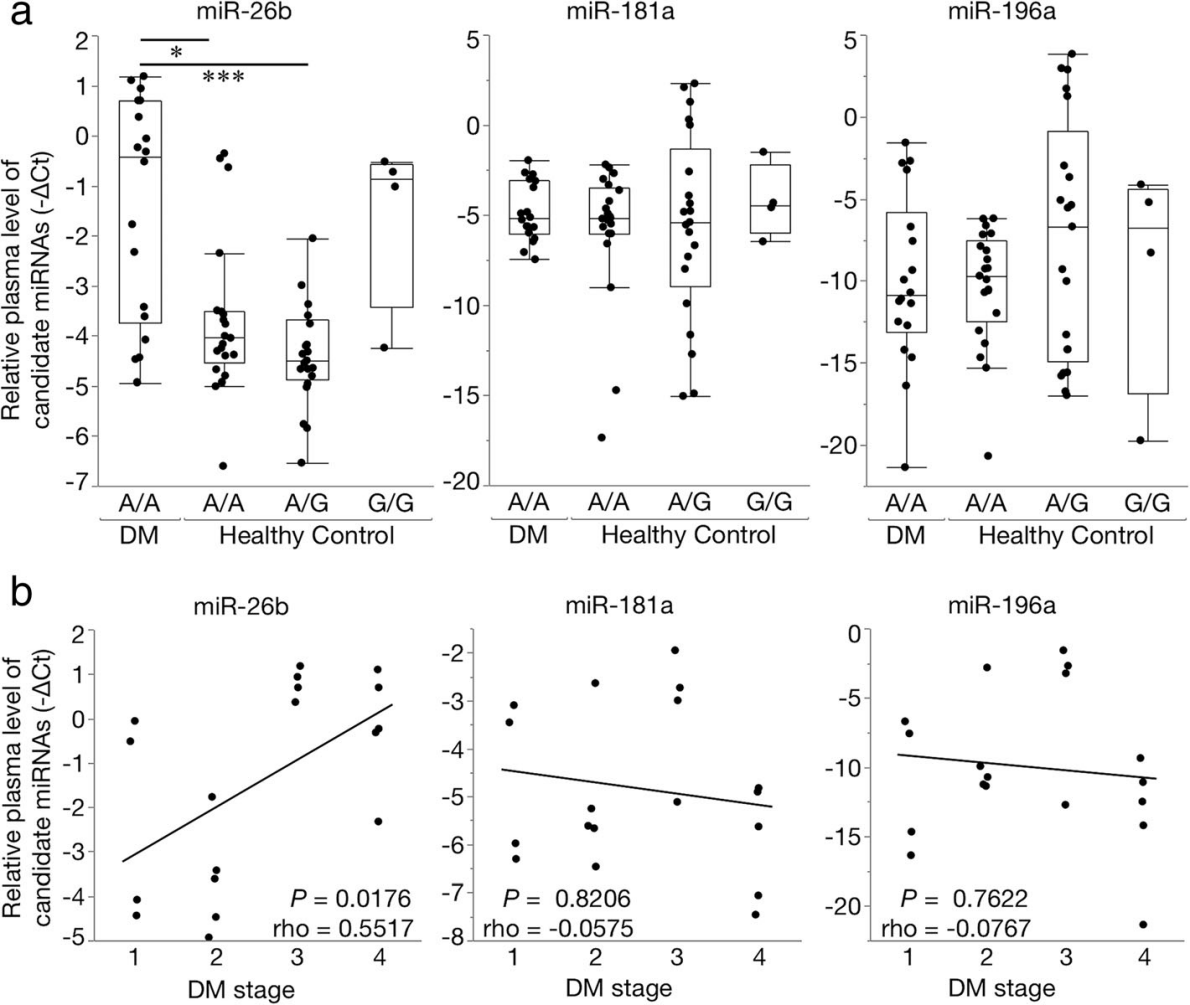
Relationship between plasma levels of candidate microRNAs (miRNAs) and clinical characteristics in an aged Pembroke Welsh Corgi (PWC) population. **a** Boxplots of candidate plasma miRNAs of degenerative myelopathy (DM)-affected PWCs and healthy control PWCs with different SOD1 alleles (Steel-Dwass test; * *P* < 0.05, *** *P* < 0.001). **b** Correlation between the relative expression levels of candidate miRNAs and DM staging (Spearman’s correlation coefficient)

The plasma level of miR-26b was significantly higher in the DM group than in healthy control PWCs carrying the A/A allele or A/G allele. On the other hand, no significant differences in the plasma levels of miR-181a and miR-196a were found between the DM group and healthy control PWCs carrying any *SOD1* allele (Fig. 5a).

In order to evaluate whether the plasma level of miR-26b correlated with DM staging, we performed a correlation analysis between the plasma level of candidate miRNAs and DM stages. A positive correlation was observed between increases in the plasma level of miR-26b and disease progression. No correlations were noted between DM staging and other candidate miRNAs (Fig. 5b).

## Discussion

In recent years, bioinformatics studies on the human genome have predicted circulating miRNAs as diagnostic biomarkers of neurodegenerative diseases, such as Alzheimer’s disease [29], Parkinson’s disease [30], and ALS [19, 21]. However, the majority of canine miRNA biomarker discovery studies have focused on neoplastic diseases, and there is currently only one study on the miRNAs of dogs with meningoencephalomyelitis of unknown origin [31]. In addition, miRNA profiles have been suggested to change with age, gender, and other environmental factors [32]. Due to its characteristics, such as breed propensity and adult late-onset, we considered DM to be the ideal disease target for discovering miRNA biomarkers. In addition, because miRNAs reflected pathological condition and are released in circulation with stable form such as microvesicles [18], miRNAs in plasma have also demonstrated their potential as non-invasive biomarkers for a wide variety of diseases. Moreover, since a definitive diagnosis of DM may only be achieved by a histopathological examination, it is clinically important to develop a premortem diagnostic biomarker of DM.

In order to discover candidate miRNAs as diagnostic biomarkers of DM, we compared the plasma miRNA profiles of DM-affected PWCs, all of which were homozygotes of the A/A allele, with those of wild-type PWCs, all of which were homozygotes of the G/G allele. G/G homozygotes were used as wild-type controls because asymptomatic dogs carrying the homozygous A/A allele or heterozygotes are known to exhibit degenerative changes in their spinal cords [8]. Our results showed that 18 plasma miRNAs were differentially expressed in DM-affected PWCs. This difference in plasma miRNA profiles indicates a failure in the regulation of RNA metabolism in DM dogs. In order to further narrow down candidate miRNAs, we performed a pathway analysis to focus on genetic networks associated with *SOD1* because the aggregation and accumulation of the mutant SOD1 protein in neurons and astrocytes have been suspected to play central roles in spinal cord degeneration [4, 6–8]. Based on the results of the pathway analysis, miR-26b, miR-181a, and miR-196a were selected.

TargetScan database suggested that miR-26b mediates APP transcription. Although the native biological roles of APP have remained elusive, APP have both neuroprotective and neurotoxic function [33, 34]. Our pathway analysis revealed that miR-26b and APP are upstream of the *SOD1* expression cascade and may indirectly mediate *SOD1* expression. In contrast, miR-181a and miR-196a, which are downstream of the cascade, may enhance *SOD1* function. The constructed pathway also contained several inflammatory mediators. Since neuroinflammation may play roles in the pathomechanism of DM [25–28], candidate miRNAs may be related to neuroinflammation. If the molecular biological pathology of DM is clarified, we may be able to select miRNAs more directly connected to the molecular pathomechanisms of DM. The present study did not evaluate direct interactions between miR-26b and its putative target genes. However, our analysis is likely to simulate the actual interaction because we constructed the network using only putative target genes showing high affinity to each miRNA in an in silico analysis.

miRNA array-based and sequencing techniques are not sufficiently sensitive to detect many miRNAs with low concentrations in bodily fluids [16]. Therefore, potential biomarkers selected by an array-based analysis need to be confirmed by RT-qPCR in isolation. In order to validate the diagnostic accuracy of the selected miRNAs, we measured the plasma levels of candidate miRNAs in aged PWCs diagnosed with DM, healthy PWCs, and IVDH PWCs as disease controls. The results obtained suggested the increase of plasma miR-26b was associated with DM-affected dogs, whereas the plasma levels of miR-181a and miR-196a were not. In clinical settings, difficulties are associated with detecting DM if the dog has other concurrent spinal cord diseases. DM often coexists with IVDH. Our results showed that the plasma level of miR-26b has potential as a diagnostic biomarker of DM with high specificity. The sensitivity of plasma miR-26b alone was relatively low, however this can be improved by combining with other diagnostic tests with high sensitivity such as genetic testing of *SOD1* mutation.

To the best of our knowledge, it has not been evaluated whether the plasma miR-26b may be the diagnostic biomarker of ALS patients. In previous reports, although miR-4649-5p, miR-4299 [35], miR-424, miR-206 [36], miR-206/miR-338-3p, miR-9/miR-129-3p and miR-335-5p/miR-338-3p [37] had a potential to be the plasma based biomarkers in ALS, there were no common miRNAs identified in our microarray study. Because these previous studies did not mention *SOD1* genetic mutation, our study may have reflected the differences of pathological mechanisms between DM and ALS or the target genes of miR-26b in dogs and humans. Clarifying the pathological roles of plasma miR-26b in dogs may lead to elucidate underlying neurodegenerative mechanisms in DM.

Dogs with the mutant *SOD1* allele do not always develop DM [12, 13]. Furthermore, dogs with not only the asymptomatic A/A allele, but also the A/G allele exhibit white matter degeneration [8]. Therefore, a genetic examination alone is not sufficient to diagnose DM. In the present study, the plasma level of miR-26b was significantly higher in the DM group than in the healthy control A/A group and A/G group. In contrast, miR-26b was not able to distinguish DM from the healthy control G/G allele. This discordance between the RT-qPCR array and individual RT-qPCR may have been caused by a small number of dogs with the G/G allele. In addition, because a positive correlation was found between the plasma level of miR-26b and DM staging, an RT-qPCR array, in which the samples used were classified as stage 3 or 4, may have overestimated the plasma level of miR-26b in the DM group. This positive correlation suggested that the plasma level of miR-26b may reflect the progression and/or prognosis of DM.

Our study had several limitations. The diagnosis of DM in the validation part of this study was not based on a histopathological examination, but on a pre-mortem clinical diagnosis. The PWCs in HC group did not have neurologic examinations and follow-up examination; therefore, it is not clear whether these dogs remain normal throughout their lifetime or develop DM later on. Furthermore, since this was a cross-sectional study, we were unable to demonstrate a relationship between temporal changes in miR-26b levels and DM stages. Dogs classified as healthy controls with miR-26b levels that exceeded the cut-off may develop DM later, in which case miR-26b may become a novel pre-symptomatic diagnostic biomarker of DM. Another limitation is that disease control was restricted to IVDH. It currently remains unclear whether other spinal cord diseases affect the plasma level of miR-26b. Moreover, the sample sizes in each group were small, particularly the G/G allele group, which did not show significant differences from other groups. However, dogs with G/G homozygosity do not develop DM, and thus, the genetic examination of *SOD1* distinguishes DM from the G/G allele without using miR-26b.

## Conclusions

Our results indicate that the plasma level of miR-26b has potential as a novel diagnostic biomarker of DM. A combination of miR-26b with a clinical diagnosis is expected to improve the pre-mortem diagnostic accuracy of DM.

## Additional files

Additional file 1: **Table S1.** Raw Ct value of PCR array. (XLSX 46 kb)

Additional file 2: **Table S2.** Relative plasma level of miRNA using RT-qPCR array in DM-affected PWCs. (XLSX 37 kb)

Additional file 3: **Table S3.** The putative mRNA targets of dysregulated miRNAs by using the TargetScan database. (XLSX 45 kb)

Additional file 4: **Table S4.** Demographic, raw Ct value and relative plasma level of candidate miRNAs. (XLSX 18 kb)

## Data Availability

All data generated or analyzed in this study are included in this published article and its supplementary information files.

## Abbreviations

ALS: Amyotrophic lateral sclerosis
APP: Amyloid beta precursor protein
AUC: Area under the curve
cDNA: Complementary DNA
CSF: Cerebrospinal fluid
Ct: Threshold cycle
DM: Degenerative myelopathy
IVDH: Intervertebral disc herniation
miRNA: microRNA
MRI: Magnetic resonance imaging
mRNA: Messenger RNA
PWC: Pembroke Welsh Corgi
ROC: Receiver operating characteristic
RT-qPCR: Quantitative reverse transcription polymerase chain reaction
SOD1: Superoxide dismutase 1

## References

[1] Averill DR (1973). Degenerative myelopathy in the aging German shepherd dog: clinical and pathologic findings. J Am Vet Med Assoc.

[2] Coates JR, Wininger FA (2010). Canine degenerative myelopathy. Vet Clin North Am Small Anim Pract.

[3] Oyake K, Kobatake Y, Shibata S, Sakai H, Saito M, Yamato O, Kushida K, Maeda S, Kamishina H (2016). Changes in respiratory function in Pembroke welsh corgi dogs with degenerative myelopathy. J Vet Med Sci.

[4] Awano T, Johnson GS, Wade CM, Katz ML, Johnson GC, Taylor JF, Perloski M, Biagi T, Baranowska I, Long S (2009). Genome-wide association analysis reveals a SOD1 mutation in canine degenerative myelopathy that resembles amyotrophic lateral sclerosis. Proc Natl Acad Sci U S A.

[5] Wininger FA, Zeng R, Johnson GS, Katz ML, Johnson GC, Bush WW, Jarboe JM, Coates JR (2011). Degenerative myelopathy in a Bernese Mountain dog with a novel SOD1 missense mutation. J Vet Intern Med.

[6] Nakamae S, Kobatake Y, Suzuki R, Tsukui T, Kato S, Yamato O, Sakai H, Urushitani M, Maeda S, Kamishina H (2015). Accumulation and aggregate formation of mutant superoxide dismutase 1 in canine degenerative myelopathy. Neuroscience.

[7] Crisp MJ, Beckett J, Coates JR, Miller TM (2013). Canine degenerative myelopathy: biochemical characterization of superoxide dismutase 1 in the first naturally occurring non-human amyotrophic lateral sclerosis model. Exp Neurol.

[8] Kobatake Y, Sakai H, Tsukui T, Yamato O, Kohyama M, Sasaki J, Kato S, Urushitani M, Maeda S, Kamishina H (2017). Localization of a mutant SOD1 protein in E40K-heterozygous dogs: implications for non-cell-autonomous pathogenesis of degenerative myelopathy. J Neurol Sci.

[9] Nardone R, Höller Y, Taylor AC, Lochner P, Tezzon F, Golaszewski S, Brigo F, Trinka E (2016). Canine degenerative myelopathy: a model of human amyotrophic lateral sclerosis. Zoology (Jena).

[10] Coates JR, March PA, Oglesbee M, Ruaux CG, Olby NJ, Berghaus RD, O'Brien DP, Keating JH, Johnson GS, Williams DA (2007). Clinical characterization of a familial degenerative myelopathy in Pembroke welsh corgi dogs. J Vet Intern Med.

[11] Toedebusch CM, Bachrach MD, Garcia VB, Johnson GC, Katz ML, Shaw G, Coates JR, Garcia ML (2017). Cerebrospinal fluid levels of phosphorylated Neurofilament heavy as a diagnostic marker of canine degenerative myelopathy. J Vet Intern Med.

[12] Chang HS, Kamishina H, Mizukami K, Momoi Y, Katayama M, Rahman MM, Uddin MM, Yabuki A, Kohyama M, Yamato O (2013). Genotyping assays for the canine degenerative myelopathy-associated c.118G>a (p.E40K) mutation of the SOD1 gene using conventional and real-time PCR methods: a high prevalence in the Pembroke welsh corgi breed in Japan. J Vet Med Sci.

[13] Zeng R, Coates JR, Johnson GC, Hansen L, Awano T, Kolicheski A, Ivansson E, Perloski M, Lindblad-Toh K, O'Brien DP (2014). Breed distribution of SOD1 alleles previously associated with canine degenerative myelopathy. J Vet Intern Med.

[14] Bartel DP (2004). MicroRNAs: genomics, biogenesis, mechanism, and function. Cell.

[15] Brites D, Fernandes A (2015). Neuroinflammation and depression: microglia activation, extracellular microvesicles and microRNA Dysregulation. Front Cell Neurosci.

[16] Sheinerman KS, Umansky SR (2013). Circulating cell-free microRNA as biomarkers for screening, diagnosis and monitoring of neurodegenerative diseases and other neurologic pathologies. Front Cell Neurosci.

[17] Tan JY, Marques AC (2014). The miRNA-mediated cross-talk between transcripts provides a novel layer of posttranscriptional regulation. Adv Genet.

[18] Mitchell PS, Parkin RK, Kroh EM, Fritz BR, Wyman SK, Pogosova-Agadjanyan EL, Peterson A, Noteboom J, O'Briant KC, Allen A (2008). Circulating microRNAs as stable blood-based markers for cancer detection. Proc Natl Acad Sci U S A.

[19] Cloutier F, Marrero A, O'Connell C, Morin P (2015). MicroRNAs as potential circulating biomarkers for amyotrophic lateral sclerosis. J Mol Neurosci.

[20] Freischmidt A, Müller K, Zondler L, Weydt P, Volk AE, Božič AL, Walter M, Bonin M, Mayer B, von Arnim CA (2014). Serum microRNAs in patients with genetic amyotrophic lateral sclerosis and pre-manifest mutation carriers. Brain.

[21] Rinchetti Paola, Rizzuti Mafalda, Faravelli Irene, Corti Stefania (2017). MicroRNA Metabolism and Dysregulation in Amyotrophic Lateral Sclerosis. Molecular Neurobiology.

[22] Heishima K, Mori T, Ichikawa Y, Sakai H, Kuranaga Y, Nakagawa T, Tanaka Y, Okamura Y, Masuzawa M, Sugito N (2015). MicroRNA-214 and MicroRNA-126 are potential biomarkers for malignant endothelial proliferative diseases. Int J Mol Sci.

[23] TargetScanHuman 7.1. Whitehead Institute for Biomedical Research, Cambridge. 2016. http://www.targetscan.org/vert_71/. Accessed 1 Dec 2016.

[24] Wikipathways. Wikipathways team, 2016. https://www.wikipathways.org/index.php/WikiPathways. Accessed 1 Dec 2016.

[25] Ivansson EL, Megquier K, Kozyrev SV, Murén E, Körberg IB, Swofford R, Koltookian M, Tonomura N, Zeng R, Kolicheski AL (2016). Variants within the SP110 nuclear body protein modify risk of canine degenerative myelopathy. Proc Natl Acad Sci U S A.

[26] Lovett MC, Coates JR, Shu Y, Oglesbee MJ, Fenner W, Moore SA (2014). Quantitative assessment of hsp70, IL-1β and TNF-α in the spinal cord of dogs with E40K SOD1-associated degenerative myelopathy. Vet J.

[27] Ogawa M, Uchida K, Park ES, Kamishina H, Sasaki J, Chang HS, Yamato O, Nakayama H (2011). Immunohistochemical observation of canine degenerative myelopathy in two Pembroke welsh corgi dogs. J Vet Med Sci.

[28] Ogawa M, Uchida K, Yamato O, Mizukami K, Chambers JK, Nakayama H (2015). Expression of autophagy-related proteins in the spinal cord of Pembroke welsh corgi dogs with canine degenerative myelopathy. Vet Pathol.

[29] Sharma N, Singh AN (2016). Exploring biomarkers for Alzheimer's disease. J Clin Diagn Res.

[30] Arshad AR, Sulaiman SA, Saperi AA, Jamal R, Mohamed Ibrahim N, Abdul Murad NA (2017). MicroRNAs and target genes as biomarkers for the diagnosis of early onset of Parkinson disease. Front Mol Neurosci.

[31] Gaitero L, Russell SJ, Monteith G, LaMarre J (2016). Expression of microRNAs miR-21 and miR-181c in cerebrospinal fluid and serum in canine meningoencephalomyelitis of unknown origin. Vet J.

[32] Silva SS, Lopes C, Teixeira AL, Carneiro de Sousa MJ, Medeiros R (2015). Forensic miRNA: potential biomarker for body fluids?. Forensic Sci Int Genet.

[33] Reinhard C, Hebert SS, De Strooper B (2005). The amyloid-beta precursor protein: integrating structure with biological function. EMBO J.

[34] Turner PR, O'Connor K, Tate WP, Abraham WC (2003). Roles of amyloid precursor protein and its fragments in regulating neural activity, plasticity and memory. Prog Neurobiol.

[35] Takahashi I, Hama Y, Matsushima M, Hirotani M, Kano T, Hohzen H, Yabe I, Utsumi J, Sasaki H (2015). Identification of plasma microRNAs as a biomarker of sporadic amyotrophic lateral sclerosis. Mol Brain.

[36] de Andrade HM, de Albuquerque M, Avansini SH, de SRC, Dogini DB, Nucci A, Carvalho B, Lopes-Cendes I, Franca MC (2016). MicroRNAs-424 and 206 are potential prognostic markers in spinal onset amyotrophic lateral sclerosis. J Neurol Sci.

[37] Sheinerman KS, Toledo JB, Tsivinsky VG, Irwin D, Grossman M, Weintraub D, Hurtig HI, Chen-Plotkin A, Wolk DA, McCluskey LF (2017). Circulating brain-enriched microRNAs as novel biomarkers for detection and differentiation of neurodegenerative diseases. Alzheimers Res Ther.

